# Beta Blockade Protection of Bone Marrow Following Injury: A Critical Link between Heart Rate and Immunomodulation

**DOI:** 10.4172/2329-8820.1000124

**Published:** 2013-06-02

**Authors:** Gregg M Baranski, Latha V Pasupuleti, Ziad C Sifri, Kristin M Cook, Walter D Alzate, Pranela Rameshwar, David H Livingston, Alicia M Mohr

**Affiliations:** 1 Department of Surgery, Division of Trauma, UMDNJ-New Jersey Medical School, Newark, NJ, USA; 2 Department of Medicine-Hematology, UMDNJ-New Jersey Medical School, Newark, NJ, USA

**Keywords:** Bone marrow, Homeostatic conditions, Norepinephrine

## Abstract

**Introduction:**

Severe trauma induces a profound elevation of catecholamines that is associated with bone marrow (BM) hematopoietic progenitor cell (HPC) colony growth suppression, excessive BM HPC mobilization, and a persistent anemia. Previously, propranolol (BB) use after injury and shock has been shown to prevent this BM dysfunction and improve hemoglobin levels. This study seeks to further investigate the optimal therapeutic dose and timing of BB administration following injury and shock.

**Methods:**

Male Sprague-Dawley rats were subjected to a combined lung contusion (LC), hemorrhagic shock (HS) model ± BB. In our dose response experiments, animals received BB at 1, 2.5, 5, or 10 mg/kg immediately following resuscitation. In our therapeutic window experiments, following LCHS rats were given BB immediately, 1 hour, or 3 hours following resuscitation. BM and peripheral blood (PB) were collected in all animals to measure cellularity, BM HPC growth, circulating HPCs, and plasma G-CSF levels.

**Results:**

Propranolol at 5 and 10 mg/kg significantly reduced HPC mobilization, restored BM cellularity and BM HPC growth, and decreased plasma G-CSF levels. Propranolol at 5 and 10 mg/kg also significantly decreased heart rate. When BB was administered beyond 1 hour after LCHS, its protective effects on cellularity, BM HPC growth, HPC mobilization, and plasma G-CSF levels were greatly diminished.

**Conclusion:**

Early Buse following injury and shock at a dose of at least 5mg/kg is required to maintain BM cellularity and HPC growth, prevent HPC mobilization, and reduce plasma G-CSF levels. This suggests that propranolol exerts its BM protective effect in a dose and time dependent fashion in a rodent model. Finally, heart rate may be a valuable clinical marker to assess effective dosing of propranolol.

## Introduction

Under normal homeostatic conditions there is a continuous flux of hematopoietic progenitor cells (HPCs) between bone marrow (BM) and peripheral blood (PB) that is influenced by the level of norepinephrine (NE) [1,2]. Following severe trauma there is a catecholamine surge, where epinephrine and norepinephrine have been shown to be markedly elevated to 2–10 times normal in both human and murine models [3–6]. This profound elevation of NE is associated with suppression of BM HPC growth and increased HPC mobilization to the PB and sites of injury [7,8]. This increase in NE also results in the alteration of the BM microenvironment with an increase in granulocyte colony stimulating factor (G-CSF) and matrix metalloprotease-9 (MMP-9), which both play a role in HPC mobilization [9,10].

Previously, the use of the non-selective beta-blocker propranolol (BB) at a dose of 10 mg/kg in rats after injury and hemorrhagic shock has prevented HPC suppression in the BM and reduced HPC mobilization [8]. In addition, propranolol use following injury and shock has been shown to decrease plasma G-CSF levels and MMP-9 [9]. Thus, in a rodent injury and shock model propranolol use following resuscitation prevents BM dysfunction and has been shown to improve hemoglobin levels seven days post-injury [8]. These observations show that propranolol may be an interesting potential therapeutic agent to prevent BM dysfunction after severe traumatic injury.

Therefore, this study seeks to investigate both the effective dose and the time of the therapeutic window for propranolol following injury and shock. Understanding the optimal dose and timing of BB after injury is essential in determining the most effective clinical application.

## Materials and Methods

### Animals

Male Sprague-Dawley rats (Charles River, Wilmington, MA) weighing 300–400 g were housed under barrier-sustained conditions and kept at 25°C with 12 hour light/dark cycles. Animal were provided ad lib access to water and food (Teklad22/5 Rodent Diet W-8640; Harlan Teklad, Madison, WI). The animal facility environment and animals were maintained in accordance with the regulations detailed in the Guide for the Care and Use of Laboratory Animals. The New Jersey Medical School Animal Care and Use Committee approved all animal protocols.

### Reagents

Sodium pentobarbital was purchased from Lundbeck Inc. (Deerfield, IL) and heparin was obtained from Hospira Inc. (Lakefront, IL). Propranolol hydrochloride (BB), bovine serum albumin (BSA), and 2-mercaptoethanol was purchased from Sigma (St. Louis, MO). Fetal bovine serum (FBS), Iscove’s Modified Dulbecco’s Medium (IMDM), glutamine, penicillin/streptomycin, and trypan blue were obtained from Invitrogen (Carlsbad, CA). Methylcellulose was purchased from Stemcell Technologies (Vancouver, Canada). All cytokines rhEpo, rhIL-3, rhGM-CSF were purchased from R&D (Minneapolis, MN).

### Experimental groups

#### A. Dose response

To study the effects of different doses of a non-selective BB, propranolol, after lung contusion/hemorrhagic shock (LCHS), animals to receive BB were given either a 1, 2.5, 5, or 10 mg/kg dose via intraperitoneal (IP) injection at a single time point, immediately following resuscitation (N=4–8 animals/group). These animals were compared to LCHS alone animals (N=10 animals/group) and an unmanipulated control (UC) group (N=7 animals/group) that did not undergo any tissue injury or shock. Animals in these groups were sacrificed at three hours following injury. To evaluate the long term effects of the various doses of BB the same rodent groups were sacrificed at day seven (N=6–10 animals/group) and the BB treated rats received their respective doses IP daily until sacrifice. Peripheral blood was acquired through cardiac puncture and BM was harvested from the left femur. Heart rate (HR) was monitored and recorded during shock, immediately after resuscitation, and at the time of sacrifice for all animals.

#### B. Therapeutic window

In order to determine the effective therapeutic window, 10 mg/kg of BB was given at various time points following resuscitation. Using the same LCHS model, we administered the BB IP either immediately, one hour, or three hours following resuscitation (N=4–5 animals/group). Animals were sacrificed at three hours following the administration of BB. In addition to UC, these animals were compared to LCHS rats (N=4–10 animals/group). Peripheral blood and BM were collected to assess BM cellularity, BM HPC colony growth, HPC mobilization, plasma G-CSF and HR.

### Combined tissue injury and hemorrhagic shock model

Experimental animals were weighed and anesthetized with IP injections of sodium pentobarbital (50 mg/kg). Unilateral lung contusion (LC) was inflicted using a blast wave percussive nail gun (Craftsman 968514 Stapler, Sears Brands Chicago, IL) applied to a 12 mm metal plate adherent to the right axilla of the rat. This model has been shown to produce a clinically significant LC as demonstrated by radiography and histology [11]. Using aseptic surgical technique, the right internal jugular vein and femoral artery were then cannulated with polyethylene (PE-50; Becton Dickinson and Co., Sparks, MD) and Silastic (Dow Corning Corp., Midland, MI) tubing, respectively. To prevent clotting, all tubing was instilled with heparinized saline (10 units/ml). The femoral artery tubing was then connected to a continuous blood pressure monitoring device (BP-2 Digital Blood Pressure Monitor; Columbus Instruments; Columbus, Ohio) for measurement of mean arterial pressure and HR. Animals were bled to a MAP of 30–35 mmHg for 45 minutes. Temperature was maintained at approximately 37°C with the use of an electric heating pad under the surgical platform. Shed blood was also maintained at approximately 37°C and was re-infused at a rate of 1 ml/min following the shock period.

### Bone marrow cellularity

BM cells were obtained from individual rats, by removing the femoral epiphysis, and aspirating the BM with an 18 gauge needle on a 5 cc syringe filled with of 1mL IMDM supplemented with 10% FBS. A suspension was prepared by passing cells through a 40 μm sterile nylon strainer to remove particulate matter. Total viable cell counts were then determined by 0.4% Trypan blue staining using a hemocytometer.

### Bone marrow clonogenic assays

Colony-forming unit-granulocyte-, erythrocyte-, monocyte-, megakaryocyte (CFU-GEMM) were used to assess the effects of LCHS ± BB on earlier progenitor cells. To specifically explore the effects on the erythroid cell lines, burst-forming unit-erythroid (BFU-E) and colony-forming unit-erythroid (CFU-E) were assessed. The normal differentiation of these progenitor cells is as follows: CFU-GEMM →BFU-E→ CFU-E→ erythrocytes [12].

Based on the cellularity, as determined using the above method, a stock solution of BM mononuclear cells was prepared to yield a concentration of 1×10^6^ cells/mL of IMDM. From this solution cultures were prepared in duplicate by removing 1.5×10^5^ cells and plating them in IMDM containing 30% FBS, 2% BSA, 1% methylcellulose, rat growth factor, penicillin/streptomycin, 2×10^−4^ mol/L 2-mercaptoethanol, and glutamine. Plates were further supplemented with either, 1.3 U/mL rhEpo and 6U/mL rhIL-3 for BFU-E/CFU-E, or 3 U/mL rhGM-CSF for CFU-GEMM. Cultures were incubated at 37°C in 5% CO_2_. CFU-E colonies were counted at day 7, BFU-E colonies at day 14, and CFU-GEMM colonies at day 17 by an observer blinded to the origin of the samples.

### Flow cytometry

We evaluated the effects BB on the percentage of circulating HPCs, defined as CD71+/CD117+ cells, by isolating these cells from whole blood via flow cytometry. The frequency of CD117+ and CD 71+ cells was quantified in unfractionated peripheral blood samples using an established, single-platform enumeration method. Briefly, 100 μl of peripheral blood (10^6^ cells) was labeled with 10 μL of BD Pharmingen^™^ mouse anti-rat CD71 antibody conjugated with fluorescein isothiocyanate and 10 μL of BD Pharmingen^™^ rat anti-mouse CD117 (c-Kit) antibody conjugated to phycoerythrin (BD Biosciences, Franklin Lakes, NJ) for 30 minutes. Following ammonium chloride erythrocyte lysis, cells were then centrifuged at 300G x five minutes and supernatant was discarded. Cells were washed three times and fixed with BD Cytofix^™^ solution (BD). Cells were analyzed using BD FACS Calibur flow cytometer (BD) equipped with Cell Quest software (BD). Samples from each group were stained and run in duplicate and an event count of 30,000 was obtained for each run. Following acquisition of data further analysis was performed using Flow Jo v.7.2.4 (Tree Star, Ashland, OR).

### Measurement of plasma G-CSF

Peripheral blood samples were centrifuged at 10,000 rpm for ten minutes at 10°C to obtain plasma, which was collected and stored at −80°C. Plasma samples were analyzed for G-CSF using commercial colorimetric sandwich ELISA kits (R&D Systems Inc., Minneapolis, MN). Assays were performed according to the provided manufacturer’s instructions. All standards and samples were assayed in duplicate.

### Statistical analysis

All data are expressed as mean ± SEM. Statistical analyses were performed using one-way analysis of variance (ANOVA) followed by Tukey-Kramer’s multiple comparison post test and Kruskal Wallis ANOVA with Graph Pad Prism (Version 4.0, San Diego, CA). Results were considered significant if *p<0.05 vs. LCHS.

## Results

### Dose response effects in bone marrow

**Figure 1:**
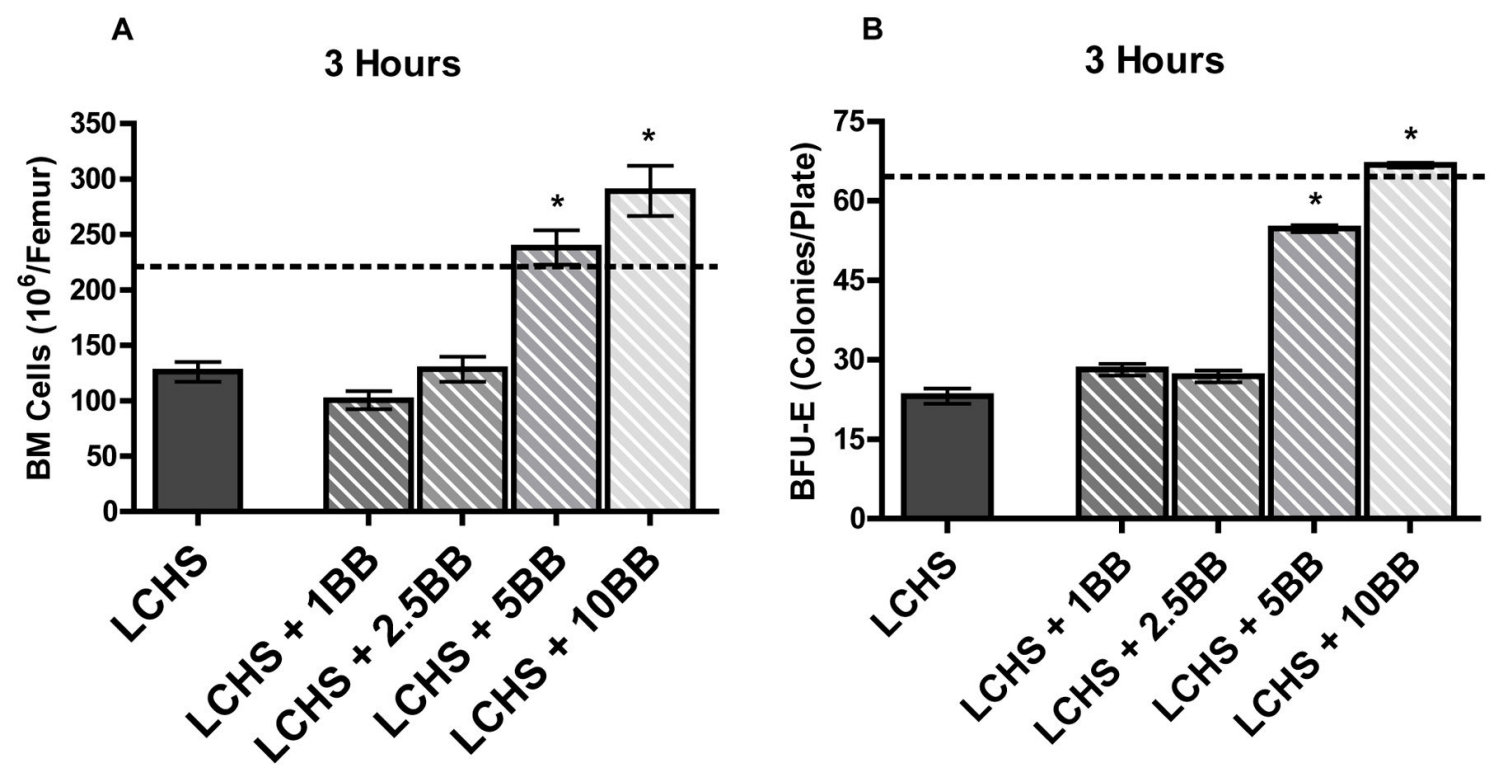
A and B. Following LCHS there is a significant suppression of BM cellularity (1A) and BM HPC growth (1B) at three hours. With the administration of BB at a dose of 5 mg/kg or higher there is preservation of BM cellularity and HPC growth. Dotted line represents UC (unmanipulated control). (n=4–10 per group). * p<0.05 vs. LCHS.

To evaluate the three hour response, BM was harvested from rats and total viable cell counts were determined for UC, LCHS, and LCHS+BB (per respective doses of 1, 2.5, 5, 10 mg/kg). Similar to our previous work, we see a 40% decrease in BM cellularity following LCHS at three hours (Figure 1A). With the administration of higher doses of 5 and 10 mg/kg of BB after LCHS, we see maintenance of BM cellularity at baseline levels. At the lower doses of BB (1 and 2.5 mg/kg), the cellularity is reduced and similar to the LCHS alone group (Figure 1A). Using BM HPC growth to assess BM function, we performed clonogenic assays, selecting for the CFU-GEMM, BFU-E, and CFU-E lines. At three hours after LCHS, there is a greater than 50% decrease in BFU-E colony growth and treatment with 5 or 10 mg/kg of BB causes statistically significant increases in BFU-E colony growth (Figure 1B). Treatment with either 1 or 2.5 mg/kg of BB offers no BM protection and BFU-E colony growth is suppressed similar to LCHS alone (Figure 1B). The CFU-GEMM and CFU-E cell lines demonstrated similar trends to BFU-E for each dose response (data not shown).

**Figure 2:**
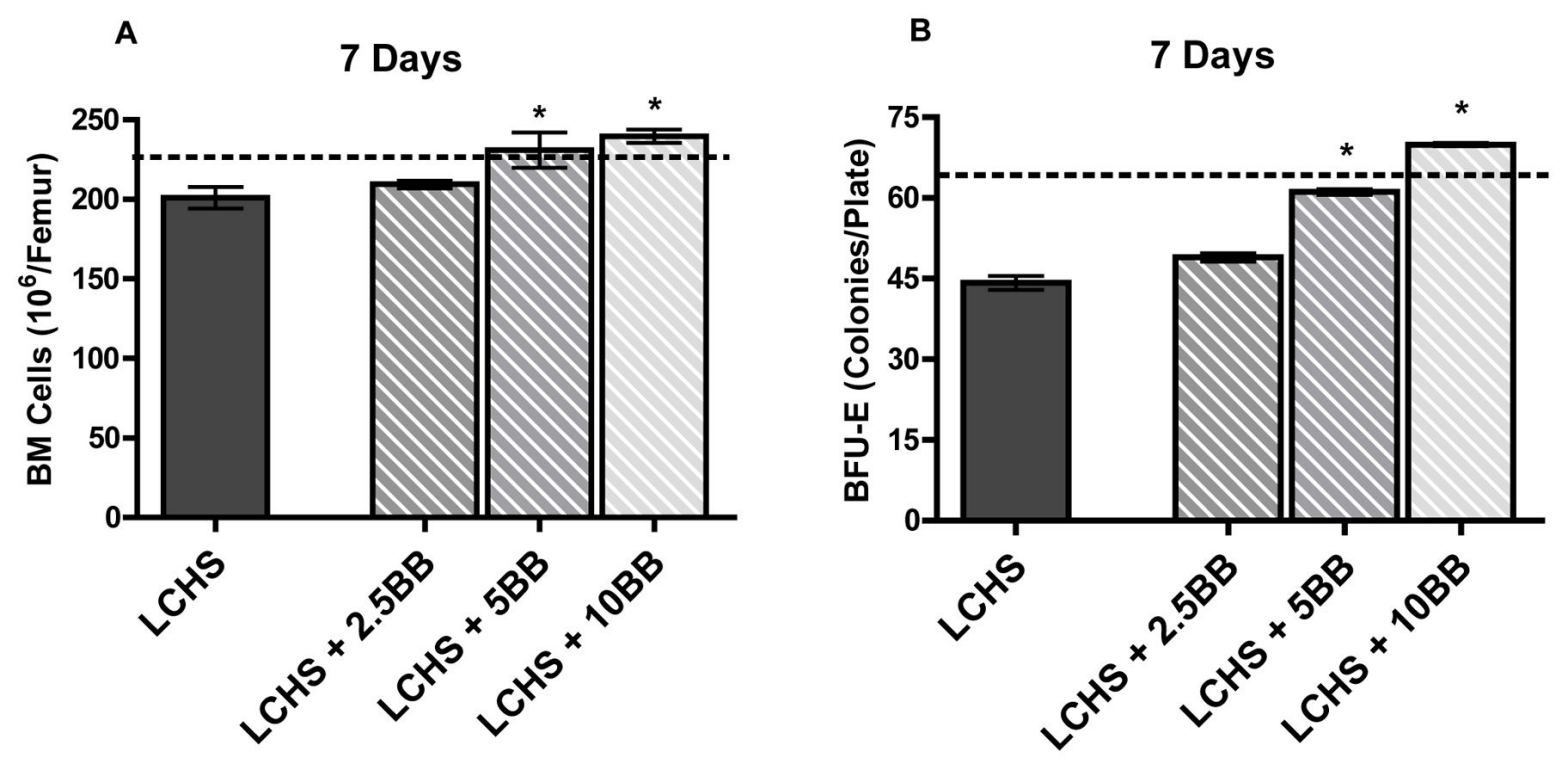
A and B. Seven days after LCHS there continues to be suppression of BM cellularity (2A) and BM HPC growth (2B) compared to control levels. Administration of BB at a dose of at least 5 mg/kg there is preservation of BM cellularity and HPC growth. Dotted line represents UC (unmanipulated control). (n=4–10 per group). * p<0.05 vs. LCHS.

Seven days after LCHS, there is still a reduction in BM cellularity when compared to UC and daily treatment with 5 or 10 mg/kg of BB after LCHS continues to significantly increase BM cellularity and protect BM (Figure 2A). Daily treatment with a lower dose of BB (2.5 mg/kg) for seven days after LCHS has no protective effect on BM cellularity (Figure 2A). Seven days after LCHS there remains a 30% decrease in BFU-E colony growth (Figure 2B). However, giving 5 or 10 mg/kg of BB preserves BFU-E colony growth and prevents prolonged BM HPC growth suppression (Figure 2B). There is no improvement in BFU-E colony growth seven days after treatment with 2.5 mg/kg of BB compared to LCHS alone.

### Dose response effects in peripheral blood

**Figure 3:**
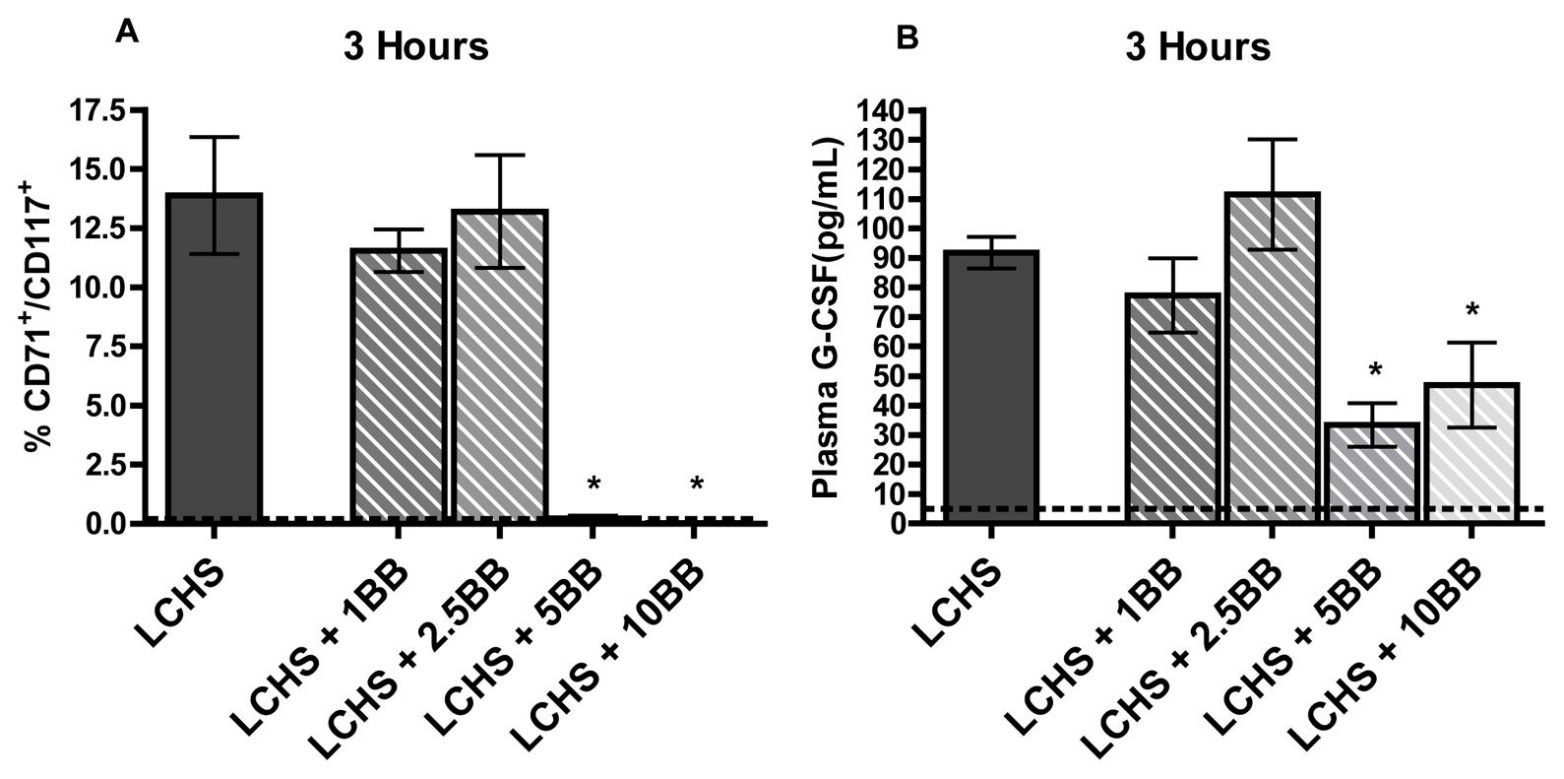
A and B. At three hours following LCHS the percentage of circulating HPCs is elevated and a dose of 5 or 10 mg/kg of propranolol prevents this mobilization (3A). Three hours after LCHS, plasma G-CSF levels are elevated and the addition of 5 or 10 mg/kg of BB significantly reduces plasma G-CSF levels. Dotted line represents UC (unmanipulated control). (n=4–10 per group). * p<0.05 vs. LCHS.

The percentage of circulating HPCs, defined as CD71+/CD117+ cells, were isolated from whole blood via flow cytometry. Figure 3A shows that at three hours following LCHS there is a significant increase in the amount of HPCs in the PB. It takes 5 or 10 mg/kg of BB to prevent HPC mobilization. Groups receiving low dose BB (1 or 2.5 mg/kg) does not prevent loss of HPCs into PB (Figure 3A). To correlate HPC mobilization, we assessed plasma G-CSF levels. Three hours after LCHS, treatment with 5 or 10 mg/kg of BB significantly reduces plasma G-CSF levels by 50% (Figure 3B). In contrast, three hours after LCHS, treatment with either 1 or 2.5 mg/kg of BB does not cause a decrease in plasma G-CSF levels (Figure 3B).

At seven days following injury, the percentage of HPCs in PB is less than 1% for all doses of BB which is similar to LCHS alone and UC animals. Similarly, plasma G-CSF levels are less than 10 pg/mL for all experimental groups seven days after LCHS. This suggests that BM HPC mobilization occurs early after injury and hemorrhagic shock in a rodent model.

### Dose response effects on heart rate

**Figure 4:**
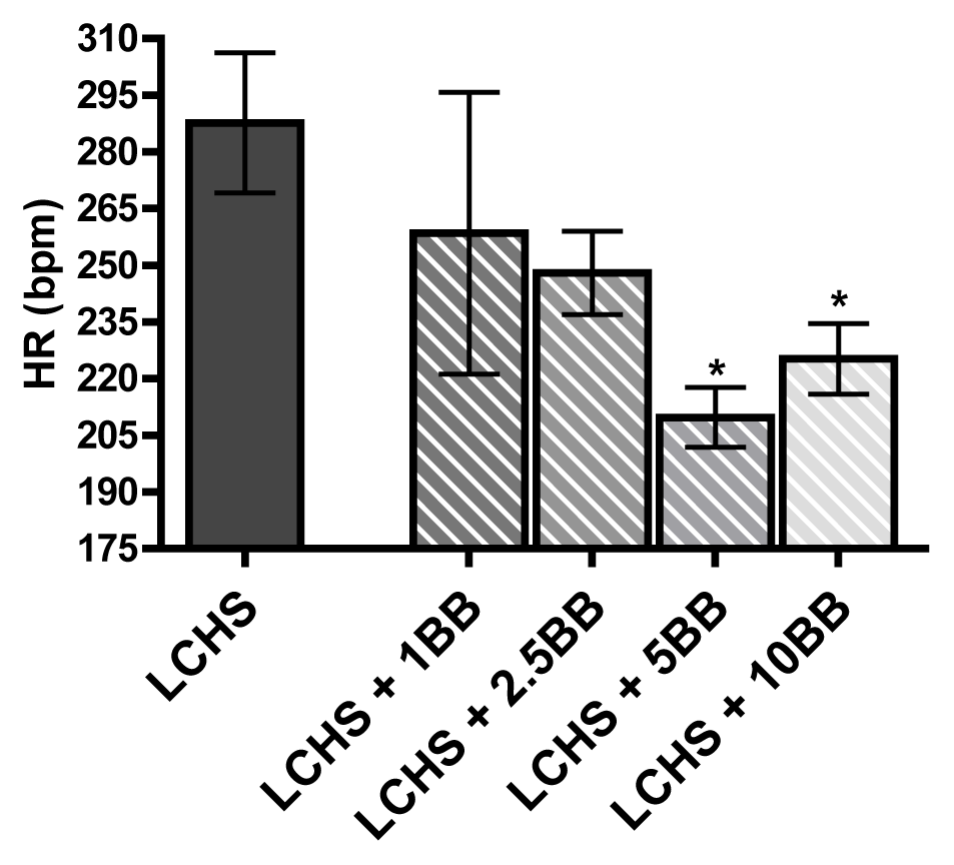
Animals that received 5 and 10 mg/kg doses of BB displayed heart rates that were significantly lower than LCHS. Dotted line represents UC (unmanipulated control). (n=4–10 per group). * p<0.05 vs. LCHS.

Average HR range in an anesthetized control animal is 300–340 beats per min (bpm). Sixty minutes after LCHS there is a slight decrease in HR to 288 ± 77 bpm. With increasing doses of BB administration, there is a reduction in HR. However, only 5 or 10 mg/kg of BB statistically reduced HR as compared to LCHS alone (Figure 4).

### Therapeutic window in bone marrow

When BB is given at or within one hour post resuscitation following LCHS, there is a preservation of BM cellularity. However, when administered beyond one hour, BB loses its beneficial effects in the BM (Table 1). Animals receiving BB at or within one hour of resuscitation show a significant increase in the growth of BM CFU-GEMM, BFU-E, and CFU-E cell lines compared to LCHS alone (Table 1). If BB is given three hours after LCHS, there is no protection of BM HPC growth (Table 1).

### Therapeutic window in peripheral blood

Animals given BB at or within one hour after LCHS showed prevention of HPC mobilization to the peripheral blood. If the animals received BB three hours after LCHS, their percentage of HPCs in PB was elevated similar to LCHS alone (Table 1). When BB was given immediately after LCHS, plasma G-CSF levels were significantly reduced. However, if BB was given one or three hours after LCHS, plasma G-CSF levels remained elevated and were similar to LCHS alone (Table 1).

## Discussion

Increasing concentrations of NE have been shown to cause a dose-dependent suppression of BM HPC colony growth in trauma and shock models both *in vitro* and *in vivo* [4,13,14]. In addition to BM HPC growth suppression, there is also a decrease in BM cellularity and an increase in HPC mobilization from the BM to the PB in rodent tissue injury and shock models [7,8,15]. The increase in HPC mobilization is also directly related to NE levels [7,8,14]. This BM dysfunction is manifest clinically as a persistent anemia lasting beyond two weeks in severely injured trauma patients [16]. In other rodent stress models, dose response effects of catecholamines are seen. In a murine rotational stress model where plasma normetanephrine, a stable end product of norepinephrine, was more than double that of control levels, these animals had delayed burn wound healing, as measured by decreased wound contracture and re-epithelialization and delayed development of granulation tissue [3]. Similarly in another murine burn model, Sivamani et al. [17] showed that after burns plasma epinephrine levels remained elevated ten times that of pre-burn levels and treatment with a beta-2 antagonist daily for ten days resulted in a significant increase in wound re-epithelialization in these animals.

Previously, we have shown that the use of propranolol following shock and injury preserves BM cellularity and BM HPC colony growth, reduces plasma G-CSF levels, and reduces BM HPC mobilization to the PB [9,15]. In addition, propranolol exerts its protective effects without delaying healing of injured tissue [7,8]. This is similar to the findings of Romana-Souza et al. [18] who showed that high dose propranolol at 25 mg/kg blocked the deleterious effects of circulating catecholamines and improved cutaneous wound healing in burned chronically stressed mice.

This study sought to further investigate the optimal dosing of propranolol necessary to produce a BM protective effect. Both 5 and 10 mg/kg of propranolol restore BM cellularity and BM HPC colony growth, and reduce HPC mobilization to PB and plasma G-CSF levels after LCHS. Lower doses of propranolol (1 mg/kg and 2.5 mg/kg) did not offer any BM protection. Given the profound elevation in catecholamines after severe trauma, it is possible that the lower doses of propranolol may not be sufficient to counteract and block the effects of circulating norepinephrine. Both 5 and 10 mg/kg of propranolol continue to be protective of BM with daily treatment seven days after LCHS. Similar dose-response effects of propranolol use have been shown in other animal models. In a rodent bone growth model, different doses of propranolol, as low as 3.5 mg/kg, were tested to measure its protective effects [19]. Propranolol only improved bone rigidity in those animals at dosed at higher doses of 7 and 10.5 mg/kg [19].

One of the most clinically relevant findings was the dose response relationship between propranolol and HR, and the correlation this had with BM protection. At the lowest doses of propranolol (1 and 2.5 mg/kg), there was no BM protection and these low doses of propranolol did not significantly affect HR following LCHS. As the dose of propranolol increased to 5 and 10 mg/kg, there was BM protection which correlated with a statistically significant decrease in HR. This suggests that HR may be a valuable marker to guide effective dosing of propranolol therapy in a clinical setting.

The BB protection of BM has been shown to be mediated via beta-2 and beta-3 receptors which are located on immune and BM cells and both the beta-2 and beta-3 receptors are involved in HPC mobilization [7]. Beiermeister et al. [7] showed that selective beta-2 and beta-3 blockade prior to lung contusion improved BM HPC colony growth and decreased HPC mobilization. In addition, Mendez-Ferrer et al. [20] demonstrated that under certain circumstances there may be cooperation of beta-2 and beta-3 receptors in BM and a double deficiency of beta-2 and beta-3 receptors significantly decreased mobilization of HPCs to the peripheral blood. Although the BM protection by propranolol is mediated by the beta-2 and beta-3 receptors, the additional effects on the beta-1 receptor and HR while not necessary for BM protection can be used to guide effective propranolol therapy. These findings are supported in burn patients where HR is currently used as a marker to guide propranolol drug therapy to reduce hypermetabolism, a noncardiac endpoint [21]. In pediatric burn patients where the chronic stress state leads to hypercatabolism, Herndon et al. [21], has been effectively using propranolol to reduce muscle protein catabolism by titrating to a dose at which there is a 20% reduction in HR. In another double blind, prospective, randomized control study by Mohammadi et al. [22], the beneficial effects of propranolol in an adult burn population demonstrated improved burn wound healing, decreased healing time, and a decreased hospital stay length of stay. Again in this study propranolol doses were titrated to decrease the patients’ resting hearting rates by 20% [22]. Our animal data correlates with the work of both these groups as there is an approximately 20% difference in HR between LCHS alone and those animals receiving 5 or 10 mg/kg of propranolol after LCHS.

In order to establish the optimal time for BB after injury, we varied the timing of propranolol administration after injury. BM suppression and egress of HPCs begins immediately after shock, and there is a narrow time window in which propranolol is effective. Administration of propranolol immediately after resuscitation or within one hour of resuscitation protects the BM. Not only is HPC mobilization prevented, but BM HPC colony growth is also protected. In a burn model, Krzyzaniak et al. [23] showed that vagal nerve stimulation post-injury protected the gut mucosal barrier only when performed within a 90 minute window from the time of injury. Early treatment after injury is also shown by Song et al. [24] who demonstrated vagal nerve stimulation following thermal injury in rats performed 30 minutes after injury provided protection from lung injury.

These findings in rodents may have significant clinical implications. While BM dysfunction after injury and hemorrhagic shock is clinically manifest as anemia and immunosuppression beyond 10–14 days after initial injury, this BM dysfunction begins immediately [16]. The elevation of epinephrine and norepinephrine beyond the initial injury period may explain the persistent anemia not attributable to the initial acute blood loss. The persistent hypercatecholanemia after severe injury is likely related to multiple patient stressors, including prolonged mechanical ventilation, sepsis, and recurrent trips to the operating room to treat multiple concomitant injuries. Based on this study and the establishment of dose and time window, it appears that early propranolol use at a dose associated with a 20% decrease in HR following severe trauma may prevent the ongoing loss of BM HPCs from the BM and BM HPC colony growth suppression. This study provides potential promise for the treatment of BM dysfunction following severe traumatic injury where no established treatment currently exists.

The results of this study establish a dose-response relationship and define the therapeutic window of propranolol for BM protective effects in a rodent model of trauma and hemorrhagic shock. In summary, early administration of propranolol at 5 or 10 mg/kg provides BM protection following injury and hemorrhagic shock. In addition, heart rate is a clinical marker that can be used to dose propranolol adequately to provide BM protection.

## Figures and Tables

**Table 1:** BB given immediately after resuscitation and at one hour maintains BM cellularity and BM HPC growth, prevents HPC mobilization, and elevation of G-CSF. (n=4–10 per group).

Therapeutic Window Bone Marrow and Peripheral Blood Parameters						
		BM HPC colony growth				
	BM Cellularity (cells x 10^6/femur)	CFU-GEMM (colonies/plate)	BFU-E (colonies/plate)	CFU-E (colonies/plate)	CD 71+/CD 117+ cells (% in PB)	Plasma GCSF (pg/mL)
UC	223 ± 12	37 ± 3	64 ± 3	72 ± 3	0.11 ± 0.07	5 ± 0.5
LCHS alone	126 ± 22	10 ± 1	23 ± 3	12 ± 3	13.88 ± 7.81	214 ± 178
LCHS+BB PR	289 ± 45*	34 ± 0*	67 ± 1*	65 ± 2*	0.04 ± 0.03*	35 ± 20*
LCHS+BB 1hr PR	227 ± 7*	31 ± 2*	56 ± 1*	60 ± 1*	0.38 ± 0.23*	176 ± 97
LCHS+BB 3hr PR	157 ± 13	13 ± 3	30 ± 4	33 ± 4	8.62 ± 1.10	215 ± 102

* p<0.05 vs. LCHS.
